# Investigation of the Wind-Direction Effect on Buoyancy-Driven Fire Smoke Dispersion in an Urban Street Canyon

**DOI:** 10.3390/ijerph20032568

**Published:** 2023-01-31

**Authors:** Kaihua Lu, Yanqing Xiang, Songyang Yu, Jie Wang, Shaohua Mao

**Affiliations:** 1 Faculty of Engineering, China University of Geosciences Wuhan, Lumo Road 388, Wuhan 430074, China; 2 China Ship Development and Design Centre, Wuhan 430064, China; 3 School of Resource and Environmental Engineering, Wuhan University of Science and Technology, Heping Avenue 947, Wuhan 430081, China

**Keywords:** smoke dispersion, urban street canyon, critical recirculation velocity, wind direction, Froude number

## Abstract

When a fire occurs in a street canyon, smoke recirculation is the most harmful factor to human beings inside the canyon, while the wind condition is an essential factor determining if the smoke is recirculated. This paper focuses on the wind direction’s effect on buoyancy-driven fire smoke dispersion in a street canyon, which is innovative research since the effect of wind direction has not been reported before. In this study, an ideal street canyon model with a height–width ratio of 1 was established, and both the wind velocity and wind direction were changed to search for the critical point at which smoke recirculation occurs. The results show that with an increase in the wind direction angle (the angle of wind towards the direction of the street width), the smoke recirculation could be distinguished into three regimes, i.e., the “fully re-circulation stage”, the “semi re-circulation stage”, and the “non-recirculation stage”. The critical recirculation velocity was increased with the increase in the wind direction angle, and new models regarding the critical wind velocity and the Froude number were proposed for different wind direction conditions.

## 1. Introduction

Due to worldwide modernization, extensive buildings and high-rise buildings are booming in cities. At the same time, the street canyon has significantly grown in modern cities, especially in commercial streets and highly dense residential areas. The concept of a street canyon was first raised by Nicholson [1] in 1975, which refers to a narrow canyon enclosed by urban streets and buildings on both sides of the street. The disturbance of high-rise buildings on both sides of the urban street on the airflow affects the removal of air pollutants, resulting in a significant decrease in the pollutant-diffusion capacity in the urban street canyon. These pollutants, aided by the wind, pose a significant risk to human health and environmental safety.

Many studies have been conducted in recent years to predict pollutant transport mechanisms in street canyons [2,3]. Pollutant flow patterns within the canyon depend strongly on the interaction between the wind flow at the top of the street and the flow around the buildings [4,5]. For free conditions without ambient wind, the flow patterns, dispersion, and transport of pollutants from vehicle emissions in street canyons were investigated by Solazzo [6], Park [7], and Zhong [8]. On the other hand, the wind effect could also be a key factor, as discussed by Oke [9]. In his study, the flow pattern within the street canyon when the incoming ambient wind was perpendicular to the street was analyzed, and the author concluded that the flow field could be categorized into three different regimes regarding different street canyon aspect ratios. The flow field in the street canyon is relevant to the external wind velocity and the configuration of the canyon [10,11,12,13,14,15]. Cui [16] studied a simulation of fire-induced pollutant dispersion in two parallel street canyons. Zhao [17] defined three fire scenarios based on the lateral location of the fire source and studied the dispersion of air pollutants caused by a fire.

Moreover, the physical structure of flow patterns can be changed due to buoyancy. The above studies only considered weak buoyancy conditions caused by solar heating or vehicle emissions; however, pollutants (e.g., the smoke generated by a fire) can also be caused by car fires [18] or tanker fires involving flammable liquids [19,20]. In these cases, there is a source of strong thermal buoyancy within the street canyon, driving the ascent of the plume, and the surrounding air would thus continuously be entrained into the fire source, leading to a more complex flow field. The flow characteristics in this case and the transport of pollutants should be determined by the competing effects of strong thermal buoyancy and the inertial effects of wind flow. Hu [21] introduced a dimensionless factor Froude number (*Fr*) to represent the relative strength of inertial force (ambient wind) upon thermal buoyancy (fire source):

(1) $$Fr=\frac{u}{\sqrt{\left(\Delta T_{z}/T_{a}\right)gz}}$$

where *u* is the ambient wind velocity, Δ*T_z_* is the local temperature rise of the plume to ambience at a vertical level of *z*, *T_a_* is the ambient temperature, *g* is the acceleration of gravity, and *z* is the local vertical coordinate (we use *z* = 18 m in this paper for the analysis of *Fr*). The buoyancy force causes the smoke to rise and exit from the top of the street canyon, but the inertial effect of the horizontal wind counteracts the buoyancy effect and tends to push the smoke to the leeward building. Due to the blockage of the leeward building, some of the smoke cannot be dispersed well but sinks down at the leeward building facade, which usually creates smoke-recirculation behavior [9]. The minimum velocity of crosswinds that causes smoke recirculation is called the “critical recirculation velocity”, denoted as *V_C_*_,_ which is a new concept for fire-induced smoke recirculation inside the street canyon. The critical recirculation velocity and Froude number were further discussed through a series of studies for different aspect ratios [22,23], with the conclusion that with an increase in the aspect ratio, the critical velocity is first increased but finally reaches a stable value, while the critical Froude number is maintained at 0.8. The effect of fire source location was also considered [21,24]. It was found that if the fire source is located at the windward building but on different floors, the critical Froude number is constant at around 0.7, but the results are different if the fire source is located at different transversal positions in the street canyon. More recently, Zhang [25] investigated the required critical wind velocity for wedge-shaped roof buildings forming different roof inclination angles, indicating that the tilt of the roof reduced the potential risk of smoke recirculation in the canyon. The influence of ambient airflow velocity and asymmetric canyon models on the dispersion of fire smoke in street canyons was reported by Wang [26].

The above studies are only focused on cases in which the wind direction is perpendicular to the street; however, there are different wind directions in practice. The wind direction is also essential to pollutant and smoke dispersion in street canyons, but it has not been fully considered in the previous literature. Even though, in many scholars’ eyes, a perpendicular wind direction subjected to a street is always representative of the worst-case scenario for pollutants, some studies have demonstrated that this is not necessarily true [27]. For instance, Soulhac and Salizzoni [28] found that when the external wind direction was parallel to the street, the pollutant concentration inside the canyon was the highest. The effect of wind conditions on the airflow and pollutant concentration field is more complex when the wind is set to an angle similar to the direction of the street [29]. Kumar [30] and Huang [31] also studied the influence of wind direction on the flow field and pollutant diffusion in urban street canyons. However, there have not been any other reports reporting the wind direction’s effect on smoke-dispersion behavior in fire scenarios with strong buoyancy-driven flows inside a street canyon.

Therefore, this paper focuses on the effect of wind direction on smoke dispersion from buoyancy-driven fires in street canyons. The modeling for this study is presented in Section 2, where Fire Dynamics Simulator software (FDS) is applied to establish an ideal street canyon model with an aspect ratio of 1. Keeping the fire heat release rate (HRR) constant at 5 MW, the critical recirculation wind velocity is obtained for different wind directions by varying the wind velocity and angle to street-direction conditions. In Section 3, the correlation of the critical recirculation wind velocity and Froude number for different wind-direction conditions are discussed, followed by a conclusion in Section 4. The research results fill a gap in the current knowledge and provide a theoretical reference for human safety in street canyons.

## 2. Modeling

### 2.1. Physical Model Configuration

**Figure 1:**
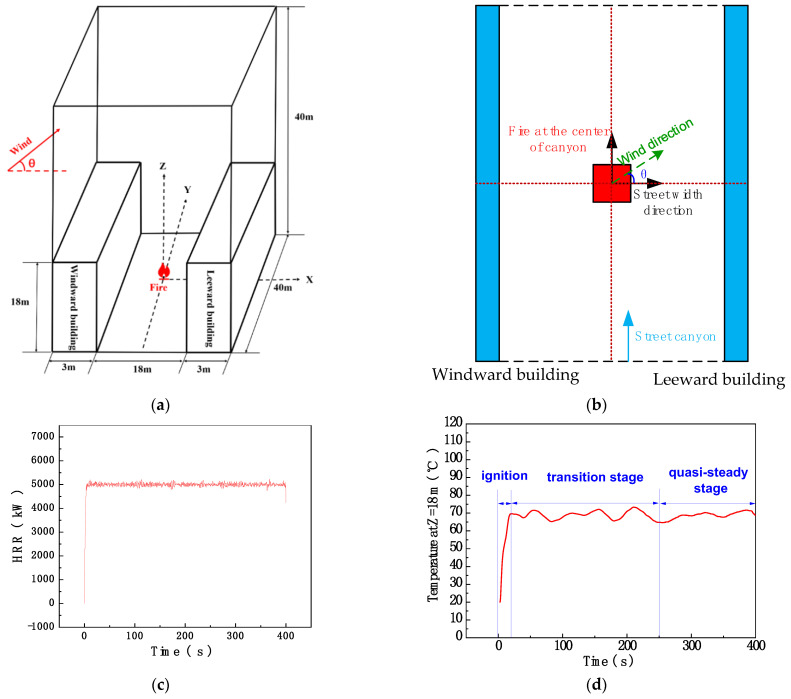
Model configuration of the street canyon under different wind-direction conditions: (**a**) general configuration; (**b**) top view; (**c**) time−based HRR curve; (**d**) time-based temperature curve for the no-wind case.

The Fire Dynamics Simulator (FDS, version 6), developed by the National Institute of Standards and Technology (NIST), was applied in this study to propose various fire scenarios with different wind conditions in a street canyon. To reduce the calculation resource load, all the simulations were processed through the large eddy simulation (LES) method in this study [32,33]. A general physical model of an ideal street canyon is displayed in Figure 1, consisting of two parallel buildings and a street between the buildings. A domain 24 m wide, 40 m long, and 40 m high was built for simulation, as shown. According to previous models [34], the two buildings were 40 m long, 3 m wide, and 18 m high, while the street was 40 m long and 18 m wide, forming an idealized street canyon (i.e., the canyon aspect ratio H/W = 1). An n-heptane fire source with a stable heat release rate of 5 MW was located at the center of the street canyon (Figure 1c), as representative of a fire caused by a traffic vehicle collision [18]. Considering the symmetry of the fire source inside the street canyon, where the wind flow could come from elsewhere, all the results are deducible and, hence, we could consider ¼ of the wind directions (i.e., *θ* ranged from 0 to 90°).

A uniform inlet velocity boundary condition was set on the left side of the simulated domain, which is similar to previous studies by Hu et al. [18], Baker et al. [34], and Zhang et al. [23]. However, the terrain upstream was not considered in this paper. The top and the other sides were deemed free boundary conditions with no initial velocity boundary condition. The bottom of the domain was set as a solid boundary. The inlet boundary conditions for the velocity field were not specified, as there is no function in FDS to set the inlet turbulence intensity value directly. However, as seen in previous studies [35], for a street canyon with an aspect ratio of 1 (W/H = 1), the FDS still predicted the flow field measurements from wind-tunnel experiments in good agreement with a uniform inlet velocity boundary condition with no specified turbulence intensity. In addition, turbulence in street canyons is not very sensitive to external turbulent conditions in the skimming flow regime, since the shear layer at the interface acts as a filter for the incoming turbulent structures [36].

To evaluate the effect of wind direction on smoke dispersion in the street canyon, a total of 14 different wind directions were considered, i.e., *θ* = 0–65° with 5° intervals, where *θ* is the angle of wind direction referred to the intersection angle of wind direction to the street width direction. In addition, the wind velocity was varied step-by-step to search for the critical wind velocity obtained from a visual inspection of the smoke-dispersion behavior over the entire simulation period. According to previous results, when *θ* = 0, the critical wind velocity was around 2.8 m/s. Physically, since the normal component of wind velocity decreases with an increase in *θ*, the critical wind velocity should increase, so the range of wind velocity was refined, as listed in Table 1. An observation slice was set up on the centerline in the Y direction to show the flow and temperature field. The initial conditions were set to an ambient temperature of 20.0 °C, ambient pressure of 1.0 atm, relative humidity of 40.0%, ambient oxygen mass fraction of 23%, and ground level of 0.0 m. The simulation duration was set to 400 s so that the situation reached a quasi-steady state [24,37] (see Figure 1d). All the simulation scenarios are listed in Table 1.

### 2.2. Grid Sensitivity

**Figure 2:**
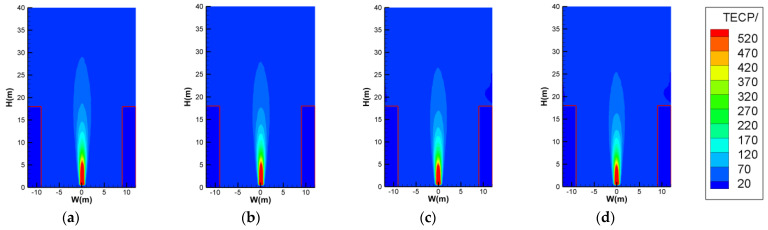

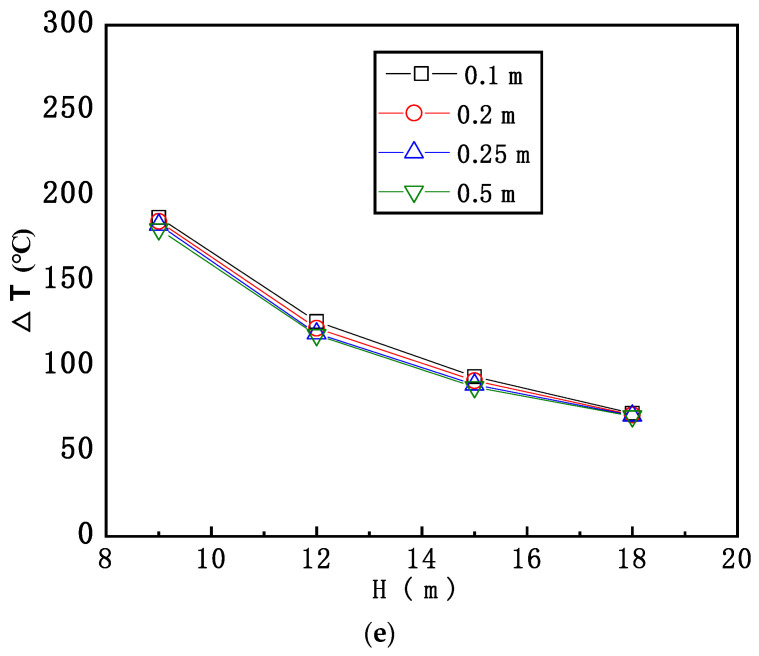
Temperature comparison for grid−sensitivity check: (**a**) grid size of 0.1 m × 0.1 m × 0.1 m; (**b**) grid size of 0.2 m × 0.2 m × 0.2 m; (**c**) grid size of 0.25 m × 0.25 m × 0.25 m; (**d**) grid size of 0.5 m × 0.5 m × 0.5 m; (**e**) temperature rise at Z = 9 m, 12 m, 15 m and 18 m above the center of fire source with different grid sizes.

Before simulation, the grid sensitivity should be discussed. Normally, the grid size δx should range from around D*/16 to D*/4 from the fire source characteristic diameter D* [38], where D* is expressed as:

(2) $$D^{*}={\left(\frac{\dot{Q}}{\rho_{\infty }C_{p}T_{\infty }\sqrt{g}}\right)}^{2/5}$$

in which $\dot{Q}$ is HRR, *ρ*_∞_ is the ambient air density, *C*_p_ is the specific heat of the air, *T*_∞_ is the ambient air temperature, and *g* is the acceleration of gravity. As for the present cases, the range of D*/16 to D*/4 was from 0.11–0.46 m (around 0.1–0.5 m), and we chose several grid sizes of 0.1 m, 0.2 m, 0.25 m, and 0.5 m for comparison to check the grid sensitivity (corresponding to 10 cells, 5 cells, 4 cells, and 2 cells within a distance of 1 m, even though 0.1 m and 0.5 m may slightly exceed the range, as indicated above). As seen in Figure 2a–d, the temperature profile at the plane of Y = 0 m was almost constant, except for a small core area of the flame (this reflects the average temperature of the finite volume so that for a smaller grid size, the average temperature should be higher). Moreover, the temperature rise at the centerline above the fire source showed good coincidence for different grid sizes (Figure 2e, with errors less than ±2.5%), so the grid-sensitivity check was successful. To reduce the consumption of calculation resources, the grid size was then set to 0.5 m.

## 3. Results and Discussion

### 3.1. Smoke-Dispersion Behavior and Flow Field Inside the Street Canyon for Various Wind-Direction Conditions

**Figure 3:**
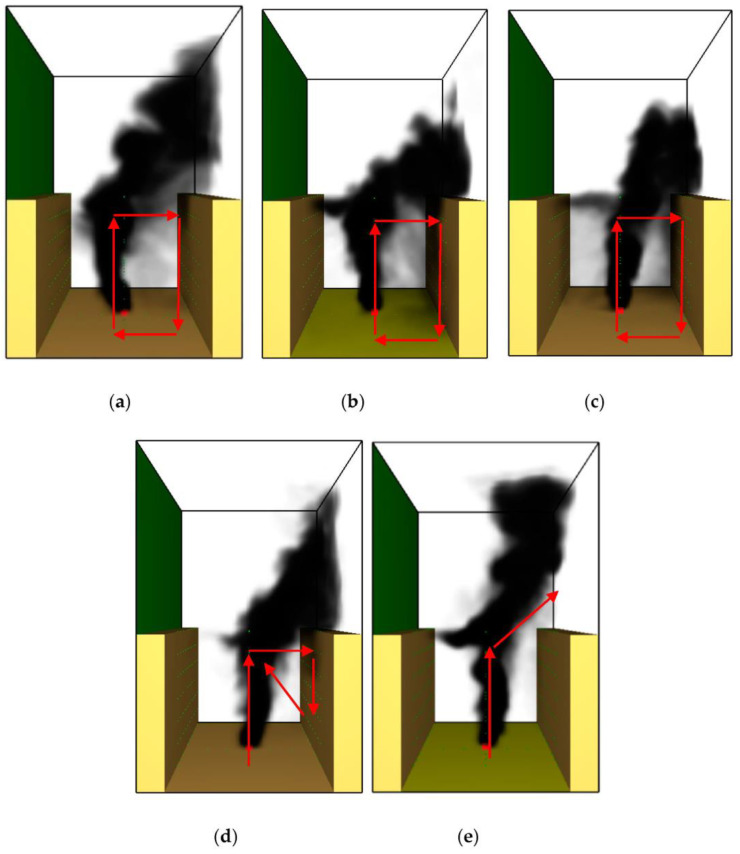
Typical 3D smoke-diffusion behavior for various wind directions at 400s: (**a**) *θ* = 0°, v = 2.8 m/s; (**b**) *θ* = 20°, v = 3.2 m/s; (**c**) *θ* = 35°, v = 3.5 m/s; (**d**) *θ* = 50°, v = 3.9 m/s; (**e**) *θ* = 65°, v = 10 m/s (**a**–**d**) refer to recirculation at a critical wind velocity, while (**e**) shows that the smoke is not recirculated).

**Figure 4:**
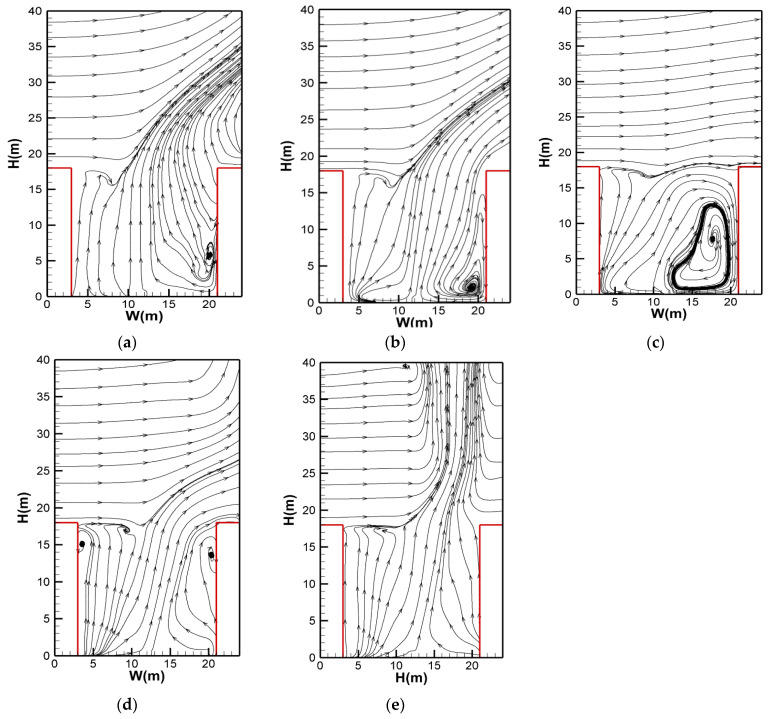
The time-averaged flow patterns for different wind-direction conditions: (**a**) *θ* = 0°; (**b**) *θ* = 20°; (**c**) *θ* = 35°; (**d**) *θ* = 50°; (**e**) *θ* = 65° (time period: 200–400 s).

The typical 3D smoke-diffusion behavior in the street canyon at 400 s for different wind directions is depicted in Figure 3. It can be seen in the figure that the smoke-diffusion behavior in the street canyon can be divided into three stages:

(1) When the angle of *θ* was small (*θ* < 35°), the smoke first spread vertically upwards due to thermal buoyancy, then tilted towards the leeward building. After impingement to the top of the leeward building, the smoke sank due to the cooling effect of the building wall, then was re-entrained by the fire source and rose up again. As time passed, the smoke filled in the entire street canyon, which is similar to previous cases with perpendicular crosswinds [18,22]. Hereby, we suggested that the recycling movement of smoke inside the street canyon be called a “fully re-circulation stage” (Figure 3a–c), which is also evidenced by the flow pattern displayed in Figure 4a–c.

(2) With the increment in *θ* (up to around *θ* = 50°), the smoke also tilted toward the leeward building, but the smoke sinking at the leeward building wall was significantly weakened. This is because (1) when the angle of *θ* is greater, more smoke is dispersed out of the street canyon through the exit in the Y direction; and (2) the distance from the smoke to the leeward building is extended (equals to $W/\left(2\cos\theta \right)$), which requires a larger inertial force to accomplish smoke recirculation. Thus, the sinking of smoke could be preserved within a short range upon the leeward building wall and eventually re-entrained at higher positions inside the street canyon. That is to say, the smoke sinking stopped near the middle–upper part of the leeward building but not at the floor, then recirculated again due to buoyancy, indicating that smoke dispersion was more likely in the “semi recirculation stage” (Figure 3d). Such “semi recirculation” behavior is shown by the flow pattern in Figure 4d, as discussed later.

(3) When the angle of *θ* was further increased, even though the wind velocity was much higher (up to 10 m/s), most of the smoke was dispersed through the canyon exit more directly. As a result, smoke sinking at the leeward building wall was rarely observed, revealing that the “non-recirculation stage” was reached (Figure 3e).

The flow patterns inside the street canyon for critical wind velocity conditions within the period of 200–400 s are presented in Figure 4, which shows good evidence of the above observations. For a relatively small angle of *θ*, a distinct clockwise vortex was formed near the leeward building (see Figure 4a–c), resulting in full recirculation inside the street canyon. As the angle of *θ* increased, the strength of the vortex was dissolved while also elevated to the upper part of the street canyon, creating a semi-recirculation behavior for the smoke (see Figure 4d). Lastly, such a vortex disappeared for a higher angle of *θ* and, hence, smoke recirculation did not take place (see Figure 4e).

### 3.2. The Effect of Wind Direction on the Critical Recirculation Velocity of Smoke

**Figure 5:**
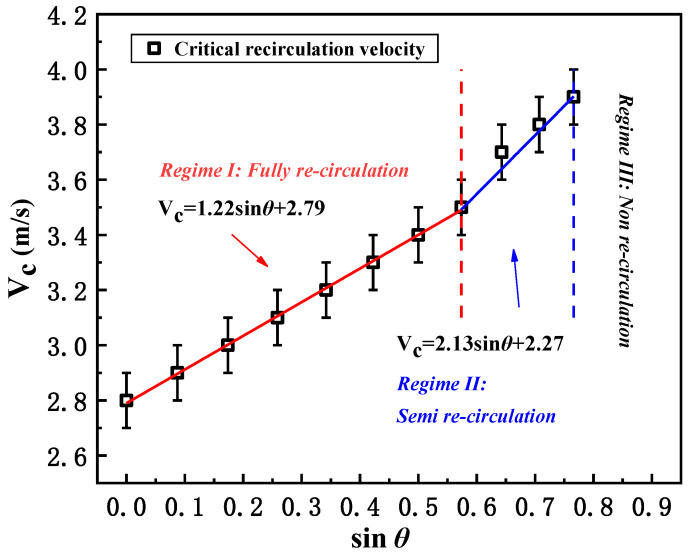
The critical recirculation wind velocity for different wind-direction conditions (the error bars are all ± 0.1 m/s regarding the potential uncertainty caused by visual inspection).

From the above, the smoke-recirculation behavior for various wind directions can be divided into three stages. As we discussed, the wind direction definitely affects smoke dispersion and, hence, the critical smoke-recirculation wind velocity can be completely different. The critical recirculation wind velocity with angles of *θ* ranging from 0° to 65° was acquired, as displayed in Figure 5. It should be noted that for the case of *θ* = 0°, in which the wind direction is perpendicular to the direction of the street, the critical smoke-recirculation wind velocity was found to be 2.8 m/s, similar to the results of Hu [18] (with HRR = 5 MW) and Wang [24]. Then, according to the different smoke-recirculation stages, variation in the critical smoke-recirculation wind velocity (with error bars ±0.1 m/s regarding the potential uncertainty caused by visual inspection) can also be categorized into different regimes (Figure 5):

(1) For the “fully re-circulation stage” (0° < *θ* < 35°), the critical recirculation wind velocity increases linearly with the angle of *θ*, which follows:

(3) $$V_{C}=1.22\sin\theta +2.79\;(\mathrm{with}\;R^{2}=0.99)$$

(2) For the “semi re-circulation stage” (35° < *θ* < 50°), there is a sudden increase in the critical velocity, and even though a linear correlation can be roughly proposed, the slope is greater than in the “fully re-circulation stage”:

(4) $$V_{C}=2.13\sin\theta +2.27\;(\mathrm{with}\;R^{2}=0.97)$$

(3) The smoke-recirculation behavior no longer takes place when the angles of *θ* exceed 50° (*θ* > 50°), referring to the “non-recirculation stage”.

Thus, as seen in the results above, when the angle of *θ* increases, the equivalent distance from the fire source to the leeward building is elongated (equals to $W/\left(2\cos\theta \right)$). This results in the promotion of critical wind velocity for smoke recirculation, which is in good agreement with Wang’s work [24]. In addition, the incline slope is higher for the “semi re-circulation stage” in Figure 5, indicating that smoke recirculation is much more difficult as the angle of *θ* exceeds 35°. The piecewise function provides the simplest way to predict *V_c_* if the angle of *θ* is determined. Moreover, the transition of “full recirculation” and “semi recirculation” could be interpreted to occur according to the different regimes of the function as it takes place at *θ* = 35° (sin*θ* = 0.574). On the other hand, we believe there may be a better circular regression method that could accurately predict the critical velocity *V_c_* by applying the correlation of *V_c_* to cos*θ*, e.g., $f={\left(\frac{\cos\theta }{1.31}\right)}^{2}+{\left(\frac{V_{c}}{4.52}\right)}^{2}=1\;\left(0.64<\cos\theta \leq 1\right)$, creating R^2^ = 0.99. However, the calculation may be more complex, and it does not reflect the transition of “full recirculation” and “semi recirculation”.

### 3.3. A New Correlation between the Critical Froude Number and Recirculation Behavior

**Figure 6:**
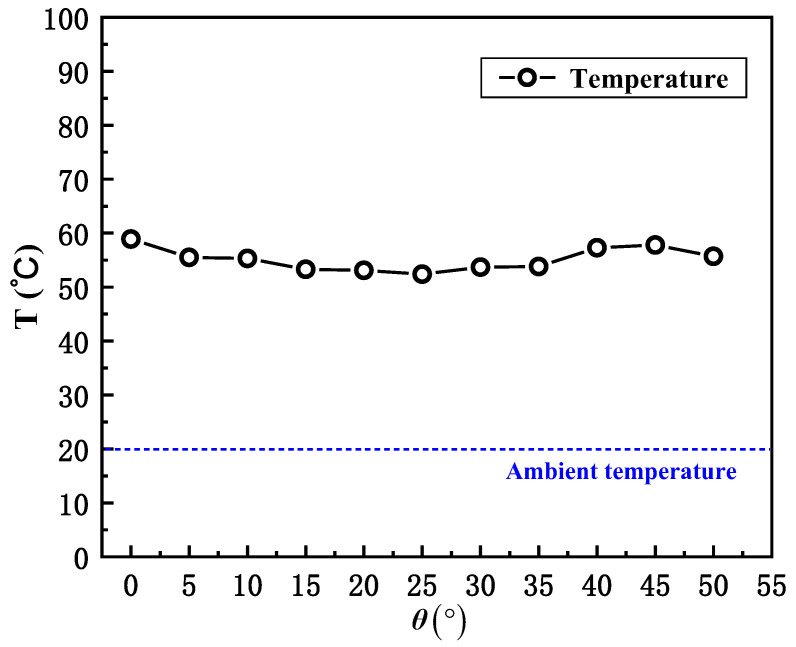
Temperature at H = 18 m above the fire source (200–400 s).

The Froude number, representing the relative strength of the inertia of ambient crosswind on buoyancy due to combustion, is an essential dimensionless factor used to characterize the smoke-recirculation behavior inside the street canyon. In particular, the Froude number is increased when the wind velocity increases or the buoyancy decreases. According to the observation slice at 200–400 s in the FDS model, there is a slight fluctuation in the temperature (55.7 °C ± 5.8%) at H = 18 m above the fire source, as seen in Figure 6. This indicates that the critical wind velocity dominates the change in the Froude number.

**Figure 7:**
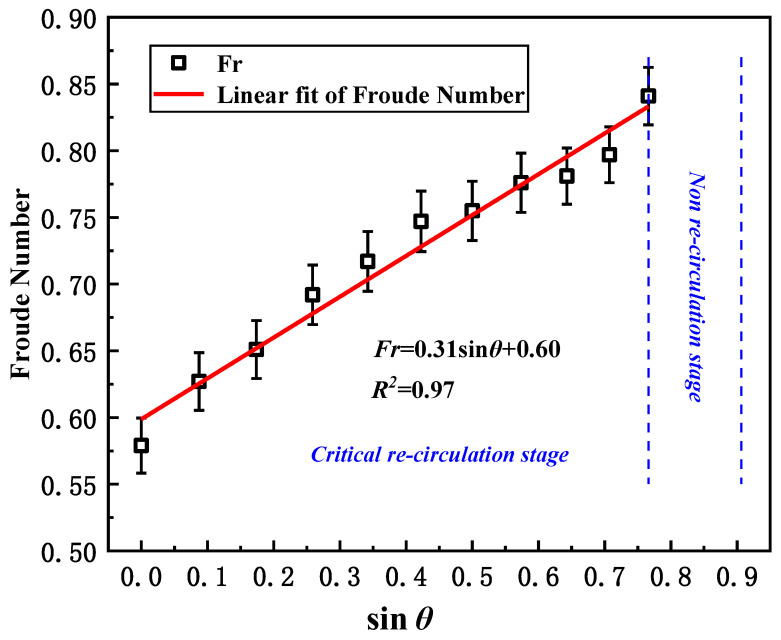
The critical Froude number in relation to various angles of *θ*.

Thus, the calculated critical Froude numbers (regarding the critical velocity of *V_c_*) for different wind direction conditions are listed in Table 2. Linear fitting is roughly achieved in Figure 7 by using the following expression:

(5)  Fr=0.31sinθ+0.60

In general, with the increase in the angle of *θ*, the inclination in the Froude number shows good accordance with the critical velocity. However, the fluctuation in temperature rise above the fire source could somewhat influence the Froude number, so only one stage could be observed. The overall correlation coefficient R^2^ is 0.97, revealing that a linear correlation is likely.

Due to the symmetry in this study, the above equations, Equations (3)–(5), provide general methods for the prediction of critical wind velocity and Froude number for smoke recirculation inside a street canyon, but it is important to note that the model is not suitable for an asymmetric canyon. Moreover, this formula does not apply when *θ* is greater than 50° because smoke recirculation is unlikely to occur when *θ* is higher. Additionally, the above model is sensitive to different HRR conditions, as the temperature difference could be higher for a greater HRR, resulting in an enhancement in buoyancy (the denominator of the *Fr*). The results apply to the ideal canyon configuration and need to be further extended in the near future.

## 4. Conclusions

This paper investigates the wind-direction effect on buoyancy-driven fire smoke recirculation behavior in street canyons. Three different stages of smoke recirculation for various wind directions were observed, and the variations in flow fields within the different stages were discussed. Then, the critical smoke-recirculation wind velocity was achieved, and a new model for the Froude number with wind direction was proposed. The main findings are as follows:

(1) The smoke-recirculation behavior of different wind directions can be divided into three different stages, i.e., the “fully re-circulation stage” for 0° < *θ* < 35°, the “semi re-circulation stage” for 35° < *θ* < 50°, and the “non- recirculation stage” for *θ* > 50°. The transition of the different stages is attributed to the smoke-diffusion modes being changed in light of different wind directions (e.g., smoke expelled from the street exit).

(2) The critical wind velocity increases with the angle of *θ*. Linear fittings can be appropriate for the critical wind velocity with sin*θ* within the “fully re-circulation stage” and “semi re-circulation stage”. Smoke recirculation is less likely to take place when the angle of *θ* is higher than 35° since there is a higher incline slope in the “semi re-circulation stage”.

(3) The Froude number gradually increases with the angle of *θ*. Even with some fluctuation in the buoyancy strength due to some minor changes in temperature, a new linear correlation is proposed for the Froude number with the factor of sin*θ* within the “fully re-circulation stage” and “semi re-circulation stage”.

This study presents the wind direction’s effect on smoke recirculation inside a street canyon, which has not been investigated before. This is of great significance for building design in modern cities and evacuation strategies for people in the area. Additionally, it is a new step forward for current models as the effect of wind direction has been taken into account. However, since the conclusions of this study are limited to a specific ideal street canyon model, we believe more factors, such as the effects of different street canyon configurations (e.g., aspect ratio, fire location, and asymmetric buildings), should be further considered in the near future.

## Figures and Tables

**Table 1:** Summary of the simulation cases.

*θ* (°)	Wind Velocity (m/s)					
0	2.6	2.7	2.8	2.9	3.0	/
5	2.6	2.7	2.8	2.9	3.0	3.1
10	2.8	2.9	3.0	3.1	3.2	3.3
15	2.9	3.0	3.1	3.2	3.3	/
20	3.0	3.1	3.2	3.3	3.4	/
25	3.1	3.2	3.3	3.4	3.5	/
30	3.2	3.3	3.4	3.5	3.6	/
35	3.3	3.4	3.5	3.6	3.7	/
40	3.4	3.5	3.6	3.7	3.8	3.9
45	3.5	3.6	3.7	3.8	3.9	4.0
50	3.6	3.7	3.8	3.9	4.0	4.1
55	3.7	3.8	3.9	4.0	6.0	10.0
60	4.0	6.0	8.0	10.0	/	/
65	4.0	6.0	8.0	10.0	/	/

**Table 2:** The critical velocity and Froude number for different wind-direction conditions.

*θ* (°)	*V_C_* (m/s)	∆*T_z_* (°C)	*Fr*
0	2.8	38.9	0.579
5	2.9	35.5	0.627
10	3	35.3	0.651
15	3.1	33.3	0.692
20	3.2	33.1	0.717
25	3.3	32.4	0.747
30	3.4	33.7	0.755
35	3.5	33.8	0.776
40	3.7	37.3	0.781
45	3.8	37.8	0.797
50	3.9	35.7	0.841

## Data Availability

The datasets used and/or analyzed during the current study are available from the corresponding author upon reasonable request.
